# The Stochastic Entanglement and Phantom Motor Execution Hypotheses: A Theoretical Framework for the Origin and Treatment of Phantom Limb Pain

**DOI:** 10.3389/fneur.2018.00748

**Published:** 2018-09-06

**Authors:** Max Ortiz-Catalan

**Affiliations:** ^1^Biomechatronics and Neurorehabilitation Laboratory, Department of Electrical Engineering, Chalmers University of Technology, Gothenburg, Sweden; ^2^Integrum AB, Mölndal, Sweden

**Keywords:** phantom limb pain, neuropathic pain, Phantom Motor Execution, virtual reality, myoelectric pattern recognition, stochastic entanglement

## Abstract

Phantom limb pain (PLP) is a debilitating condition common after amputation that can considerably hinder patients' quality of life. Several treatments have reported promising results in alleviating PLP. However, clinical evaluations are usually performed in small cohorts and rigorous clinical trials are scarce. In addition, the underlying mechanisms by which novel interventions alleviate PLP are often unclear, potentially because the condition itself is poorly understood. This article presents a theoretical framework of PLP that can be used as groundwork for hypotheses of novel treatments. Current hypotheses on the origins of PLP are discussed in relation to available clinical findings. Stochastic entanglement of the pain neurosignature, or connectome, with impaired sensorimotor circuitry is proposed as an alternative hypothesis for the genesis of PLP, and the implications and predictions this hypothesis entails are examined. In addition, I present a hypothesis for the working mechanism of Phantom Motor Execution (PME) as a treatment of PLP, along with its relation to the aforementioned stochastic entanglement hypothesis, which deals with PLP's incipience. PME aims to reactivate the original central and peripheral circuitry involved in motor control of the missing limb, along with increasing dexterity of stump muscles. The PME hypothesis entails that training of phantom movements induces gradual neural changes similar to those of perfecting a motor skill, and these purposefully induced neural changes disentangle pain processing circuitry by competitive plasticity. This is a testable hypothesis that can be examined by brain imaging and behavioral studies on subjects undergoing PME treatment. The proposed stochastic entanglement hypothesis of PLP can be generalized to neuropathic pain due to sensorimotor impairment, and can be used to design suitable therapeutic treatments.

## Introduction

Pain is an integral part of our sensory repertoire and a necessary alarm system that, when functioning normally, protects our body from harm. Unfortunately, faults in the neurological system can result in malign pain that persists despite the absence of tissue damage, namely neuropathic pain. Neuropathic pain serves no apparent biological purpose and can considerably hinder the quality of life of those it afflicts. Phantom limb pain (PLP) is one of such neuropathic pains arising from the loss of an extremity. PLP is the most common problem faced by amputees (1), and it can appear independently of the cause of amputation (2). It can begin soon after amputation and does not often diminish over time (3), thus becoming a chronic condition resistant to treatment. Amputees with PLP are less likely to use a prosthesis resulting in further disability (4). PLP worsens with situational stress (5), and most amputees report intrusion of PLP during sleep, as intense pain episodes can wake sufferers multiple times throughout the night (6). This feeds into a vicious cycle since disrupted sleep has been found to reduced pain tolerance (7). Pain itself is a multidimensional experience. Stress and depression affect perception of PLP, but do not appear to cause it (8). In addition, neuropathic pain is less understood than nociceptive pain, which causes further complications as humans are known to be less empathic to those suffering from poorly understood conditions (9). Cervero provides a comprehensive description of known pains accounting for the sensory, emotional, and cognitive components of the pain experience (10).

This article presents known hypotheses on PLP in relation to current clinical findings and the challenges they present to the theoretical frameworks upon which PLP treatments are based. Here, I propose an alternative hypothesis for the genesis of PLP that accounts for discrepancies in previous ideas on the origin of PLP, namely, the stochastic entanglement of pain with susceptible sensorimotor circuitry. The implications of and predictions made by this hypothesis are discussed in relation to clinical and neuroscientific literature. In addition, a second hypothesis is here presented for the working mechanisms of a novel treatment that has shown promising results in patients with chronic intractable PLP, namely Phantom Motor Execution (PME) (11). Current hypotheses and treatments of PLP are addressed first, foregrounding a theoretical framework for the stochastic entanglement hypothesis of PLP, and potential working mechanism of PME.

## Defining the problem

Multiple and, at-times, conflicting definitions of PLP exist. The International Association for the Study of Pain (IASP) defined PLP and stump pain during its global year against neuropathic pain (2014–2015) as follows:

Phantom limb pain is pain perceived as arising from the missing limb

Stump pain is pain perceived in the stump or residual limb

These definitions focus on the source of perceived pain, but encompass at least two different mechanisms for pain perception, namely, nociception, and neuropathic pain. I gesture to this distinction by referencing nociceptive and neuropathic PLP as follows:

*Neuropathic Phantom Limb Pain is pain perceived as arising from the missing limb due to sources other than stimulation of nociceptive fibers that used to innervate the missing limb*.

*Nociceptive Phantom Limb Pain is pain perceived as arising from the missing limb deterministically by stimulation of nociceptive fibers*.

The word “deterministically” implies that pain perception can be linked to given stimuli. Nociceptive pain in this case often corresponds to neuroma pain. Excitation of the neuroma produces afferent discharges in nociceptive fibers, which results in painful sensations perceived in the phantom limb, as said fibers previously innervated the missing limb. Bacteria could also stimulate nociceptive fibers (12), and thus elicit distally referred painful sensations in the phantom. Similarly, a viral infection can trigger PLP years after amputation, and then recede with the treatment of the infection (13). In such a case, efforts to alleviate PLP using cognitive therapies while disregarding the infection would be inappropriate and rather futile.

PLP is a complex condition that requires careful evaluation (14). Treatments for nociceptive and neuropathic pain differ, and rightly so, owing to the differences in their underlying mechanisms. A distinction between the underlying origins of pain, in addition to its location, is thus critical to help clinicians and researchers attend to the different sources of referred painful sensations (15). In addition, distinct pains are studied separately in scientific inquiry, and although a holistic approach to pain is normally recommended, clarity on the underlying causes of painful sensations perceived in the missing limb can better serve physicians in their treatment, thus improving care. Terms such as neuroma pain are already used clinically to describe nociceptive PLP, differentiating it from neuropathic PLP (16, 17). In the spirit of clarity, the term phantom limb pain is reserved for non-nociceptive pain hereafter under the following definition:

*Phantom limb pain is pain perceived as arising from the missing limb due to sources other than stimulation of nociceptive neurons that used to innervate the missing limb*.

## Theoretical framework on phantom limb pain

Different theories of pain exist, but as of yet no single theory can account for all of pain's complexity (18). Melzack's ideation of a pain *neurosignature* provided a conceptual framework referring to the particular patterns of brain activity related to pain perception (19). Under his *neuromatrix* theory of pain, Melzack proposed that the multidimensionality of a painful experience resides in a widely distributed neural network, and it is the activation pattern in said network that culminates in the perception of pain. This implies that pain perception requires more than noxious sensory input; rather, it necessitates that such input activates the pain *neurosignature*. Furthermore, sensory input is not the only way to activate said pain *neurosignature*, as in the case of neuropathic pain. More recently, the idea of pain *neurosignature* has been further refined as the *dynamic pain connectome*, describing the “*spatiotemporal signature of brain network communication that represents the integration of all aspects of pain*” (20). Kucyi and Davis proposed this concept in effort to account for fluctuations in pain perception due to attention (20).

It is worthy of notice that circuitry in the spinal cord and peripheral nerves feed into the behavior of such a pain *neurosignature* (or *connectome*), and plasticity at this level might contribute to PLP (14, 21). More importantly, it is yet unclear how said pain *neurosignature* entangles with non-nociceptive circuitry resulting in its activation, despite the absence of tissue damage or of a limb itself. These gaps are not satisfactorily addressed, if at all, by the following most prominent ideas of the genesis of PLP.

### Current ideas on the origins of phantom limb pain

#### Peripheral nociception

**Figure 1:**
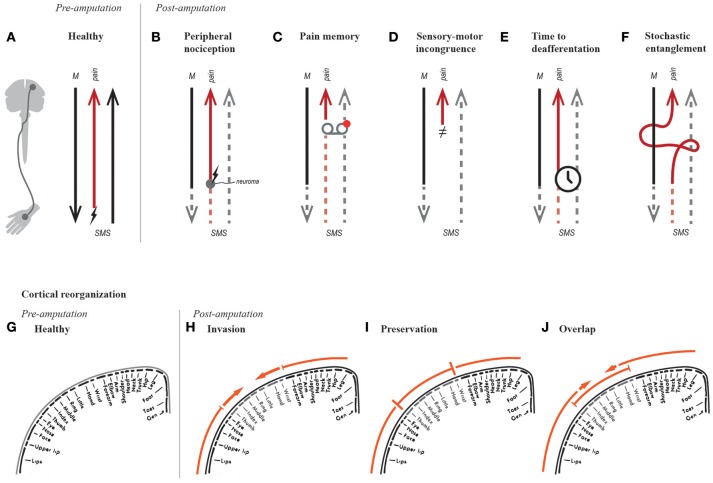
Hypotheses on the genesis of phantom limb pain. Simplified schematic of motor (“M”) and somatosensory (“SMS”) signals in a healthy able-bodied, and a person post-amputation. The red arrow resulting in pain perception represents the entire pain neurosignature along with nociceptive fibers where relevant **(A–F)**. For instance, in a healthy subject, the red signal represents nociceptive afferents firing when stimulated (“↯”), as well the neurosignature that results in pain perception **(A)**. The hypotheses of PLP are illustrated in function of changes in efferent and afferent pathways **(A–F)**, as well as their processing circuitry, such as sensorimotor cortical representations **(G–F)**.

Stimulation of nociceptive fibers produces distally referred painful sensations, similar to the way stimulation of afferent fibers once connected to lost mechanoreceptors produce distally referred tactile sensations (22). PLP was initially thought to be related to ectopic nociceptive activation at the neuroma (Figure 1B), and therefore initial treatments targeted the dissection or prevention of neuroma formation, unfortunately demonstrating limited success with relieving PLP (23). Peripheral nociception accounts for referred sensation originating at the neuroma, and thus is more appropriately called neuroma pain, rather than PLP. As previously noted, infections can also trigger nociceptive fibers (12, 13), and these can be dealt with using the appropriate antimicrobial agents.

#### Pre-amputation pain precludes PLP (“pain memory”)

Observational bias along with an intuitive understanding of memory led to the popular belief that pre-amputation pain often translates to PLP post-amputation (Figure 1C). Although this relationship has been reported (24), recent studies have found no correlation between pre-amputation pain and PLP (3, 25–27). This led Nikolajsen and Jensen to conclude that PLP is hardly preventable pre-operatively (26). As with many discussions on pain, some scientists would argue that this matter remains unsettled. Nevertheless, adequate pain management prior to amputation is recommended, firstly because unnecessary suffering must be prevented, and secondly because sustained pain should be avoided in the case that it is indeed a source driving maladaptive plasticity.

#### Sensory-motor incongruence

In 1999, Harris proposed that neuropathic pain might be caused by incongruence between motor intention, awareness of movement, and visual feedback (28). He made the intuitive analogy with motion sickness as caused by incongruent input from the visual and vestibular systems. In the case of PLP, the absence of a limb results in missing proprioceptive and visual feedback when the subject intends to move the lost limb (Figure 1D). Despite recognizing the role of proprioception, Harris placed notable importance on the therapeutic effect of visual feedback, suggesting that treatments prioritizing it would have higher chances of successfully relieving PLP.

#### Cortical reorganization

Flor et al. have provided considerable evidence on the correlation between PLP and reorganization of primary sensory and motor cortices (29–32), particularly on the activation of an area originally corresponding to the missing limb by neighboring body parts (“invasion” became a synonym of cortical reorganization, Figure 1H). They proposed that shifts in cortical representation could represent a potential neurophysiological basis for PLP (29). A causal relationship was then suggested after observing that reduction of PLP was accompanied by a normalization of cortical representation (reduced invasion), albeit in a small number of patients (33). In addition, somatosensory cortical reorganization has been observed in other conditions such as complex regional pain syndrome (CRPS) (34), and carpal tunnel syndrome (35). Reduced cortical reorganization (invasion) along with decreased PLP (36, 37) or CRPS (38) has been observed after motor or sensory training. Nevertheless and despite numerous studies, scientific evidence supporting the relationship between PLP and cortical reorganization, observed by functional brain imagining, was considered limited in a systematic review by Jutzler et al. (39).

#### Reduced functional connectivity

In recent years, Makin et al. have challenged the correlation between PLP and cortical reorganization (40, 41). They found that subjects with PLP had preserved cortical representations (Figure 1I), as opposed to cortical reorganization (Figure 1H). In addition, they found a correlation between reduced inter-hemispheric functional connectivity and PLP (40). The apparent discrepancy between cortical reorganization and preservation has been resolved by Raffin et al. who examined the cortical representation of the missing limb and both neighboring body parts (42). They concluded that activation of the missing limb and adjacent body parts can overlap, and thus invasion and preservation can coexist (Figure 1J). This finding preserves the relevance of the term “cortical reorganization” in describing functional changes in the sensory and motor cortices. The secondary finding from Makin et al. regarding the correlation between reduced functional connectivity and PLP, seems to be supported by behavioral observations of PLP accompanying reduced bimanual coupling (43, 44). Therefore, the idea of reduced functional connectivity as neural correlate of PLP is worthy of further consideration.

#### Time to deafferentation

The speed at which deafferentation and motor impairment occurs might be more relevant to the genesis of PLP than the maladaptive neural changes themselves (e.g., cortical reorganization and reduced functional connectivity) (Figure 1E). This idea was supported by Simmel, who found no presence of phantom limbs in 18 subjects who experienced slow, progressive loss of an extremity (45). However in 1976, Price examined 42 patients with leprosy and concluded that speed at which their extremities were lost, or the loss itself, did not influence the presence or absence of phantom limbs; rather, sensorimotor impairment was enough (46). For reasons unclear, this study by Price is often cited incorrectly to support the argument that gradual deafferentation does not result in the appearance of phantom limbs, and by consequence PLP.

## The stochastic entanglement hypothesis for the neurogenesis of PLP

### Explanatory challenges of the current ideas on the origins of PLP

The findings and ideas by committed scientists around the world in the past decades, prominently by Melzack et al., Flor et al., Ramachandran et al., and others, have sparked great interest in PLP, inspiring new treatments and approaches to its study. As knowledge about the condition grows, some of those ideas are validated, dismissed, or complemented by new findings. It is worthy of mentioning that even when dismissed, hypotheses on PLP have enriched our understanding of the condition. This manuscript presents arguments supporting or challenging current ideas on the genesis of PLP based on clinical observations.

The concepts of cortical reorganization and functional connectivity have in common the appearance of maladaptive changes due to the loss of sensory input and motor control. Whereas both of these hypotheses had focused on brain circuits, maladaptive plasticity in the spinal cord could also be responsible for maintaining PLP (14, 21). Hereafter, I refer to the ensemble of these changes as maladaptive neural changes, which includes both brain and spinal circuitry.

The sensory-motor incongruence hypothesis has the downside of untestability, as it can hardly be isolated from the loss of sensory input and the neglect of motor output, both of which could drive maladaptive neural changes. Strictly speaking, one would have to restore both near-natural control and sensory feedback in order to truly resolve sensory-motor incongruence. This in turn would restore the original cortical maps and increase inter-hemispheric communication, hence resolving PLP due to these primary consequences rather than due to sensory-motor congruence in and of itself.

One could argue that sensory-motor incongruence also exists at the root of pain after motor impairment, as is the case in spinal cord injuries. Spinal cord injury patients preserve their biological limbs, but sensory input and motor output is limited or nonexistent. Pain driven by a process of sensory-motor incongruence would require that the subject intends to produce movements without an appropriate sensory response. However, patients with motor impairment quickly learn that motor intention is futile and stop trying, and without movement, there is no sensory incongruence. Therefore, the appearance of referred pain years after injury or amputation, once “learned paralysis” has been established, does not correspond with the neurogenesis of pain this hypothesis suggests.

PLP can appear immediately after amputation or several years later (24, 47, 48). This temporal variation poses an additional challenge for the *sensory-motor incongruence* hypothesis. Immediate appearance of PLP would indicate that a remarkably short time is required for the sensory-motor mismatch to induce pain, which in principle could be reproduced, and thus verified, in acute laboratory experiments. On the other hand, PLP onset years after amputation would indicate that establishing sensory-motor incongruence is a relatively slow process, which contradicts the previous case (appearance of PLP immediately after amputation). A long period between PLP onset and amputation also undermines the *time to deafferentation* hypothesis.

### Stochastic entanglement of pain and somatosensory-motor circuitry

The aforementioned explanatory shortcomings aside, the major gap in the sensory-motor incongruence, cortical reorganization, and reduced connectivity hypotheses is the actual linkage of pain perception with the observed neurophysiological responses after amputation. Here, I argue that, after amputation or sensorimotor impairment, the related motor and somatosensory circuitry (cortical and sub-cortical) falls into a susceptible state of perturbation and wiring to other networks or *neurosignatures*, such as that of pain perception. In a chaotic network state of somatosensory and motor deprivation, **stochastic entanglement** can occur between networks of sensorimotor processing and pain perception (Figure 1F), which otherwise would be activated together exclusively due to noxious stimuli.

Current ideas on the genesis of PLP do not account for patients who do not develop it. All amputees experience sensory-motor incongruence, but not all develop PLP. Furthermore, not all PLP sufferers demonstrate cortical reorganization, and, conversely, not all patients with cortical reorganization develop PLP. Chaos theory has shed light on the behavior of complex dynamic systems, where small variations in initial conditions can yield different outcomes (49). The human brain is a complex dynamic system, probably the most complex system we have ever attempted to study. It is inherently noisy, but such noise arguably gives rise to its remarkable stability (50). However, a major traumatic event, such an amputation, could yield instabilities in which stochastic firing in close proximity networks (coinciding temporally and spatially), could link these networks together. Emotional and cognitive responses to such previously inconceivable perception could then enforce said link (51, 52). The stochastic nature of this process would account for the observed vicissitudes of PLP: its incidence, its degrees of intensity and repertoire of qualities, and its temporal onset after amputation. In addition, since stochastic entanglement can take place at both cortical and sub-cortical levels, it can account for the resulting alterations in the brain and spinal cord.

The experience of pain is embodied, meaning that it is always perceived in a location of the body mapped in the somatosensory cortex, which also processes other sensory percepts and it is closely linked to motor control. Therefore, circuitry for sensorimotor processing and pain perception is already linked as observed in nociceptive pain, but this relation remains selective to noxious stimuli in healthy subjects. This is despite their possibly sharing of neural resources. Furthermore, most neurons receive input from several other neurons, but have preferential activation for a subset of them. In the stochastic entanglement hypothesis, the aforementioned selectivity and preferred activation of the pain *neurosignature* is modified due to stochastically synchronized firing between the sensorimotor and pain networks. Spurious synchronized activations of neurons belonging to these networks would normally be inconsequential, but malignly established given the altered stated of sensorimotor deprivation after limb loss.

Purposely performed training of a certain skill gradually induces brain changes that do not lead to pain, as in the case of increased auditory cortical representation in musicians (53). More specifically to motor cortex, string players have shown an enlarged representation of the left hand digits proportional to the time they began playing (54). In amputees, sensory discrimination training has shown to enlarge the stump somatosensory representation while also reducing PLP (55). These examples contrast the known correlation of PLP with uncontrolled and unpurposeful cortical reorganization (29–32). I argue that the presence of such brain changes is not as important as the chaotic state in which they occur, because this is what can potentially allow the entanglement with the pain *neurosignature* or *connectome*.

### Treatments and predictions resulting from the stochastic entanglement hypothesis

Restoration of motor control and sensory feedback is the ideal treatment for PLP as suggested by all plasticity-based hypotheses. Strictly speaking, sensory-motor incongruence can only be resolved by the aforementioned two-fold restoration (sensory and motor). Similarly, the ideal solution based on cortical reorganization would be to reverse it by restoring motor and sensory maps. However, the possibility exists that by restoring either motor or sensory impairment, one can still normalize cortical changes to a certain extent. This is because activity of the sensory and motor cortices is highly interlinked, to the point that findings on the active role of the sensory cortex in motor control have called for reevaluation of the functional organization of cortical maps (56). The stochastic entanglement hypothesis proposed here suggests that in addition to the aforementioned solutions, purposeful enlargement of the stump representation in the cortex could also alleviate PLP. In other words, cortical reorganization without resultant PLP is possible under the hypothesis of stochastic entanglement, given that such reorganization happens in a gradual and functionally driven (purposeful) manner. Since stochastic entanglement can be conceived as a function of Hebb's law, “*neurons that fire together, wire together*”, PLP relief could be achieved by the same law's inverse “*neurons that fire apart, wire apart*.” Once sensorimotor and pain circuitry have entangled, one could disentangle them by repeatedly activating one without activating the other, thus weakening their connection. Repetitive recruitment (native or repurposed) of the affected sensorimotor circuitry is thus an avenue for treatment of PLP based on the stochastic entanglement hypothesis.

Literature citing neuroplasticity-based hypotheses of PLP repeatedly emphasizes the need for anthropomorphic visual feedback to alleviate PLP (57, 58). Harris predicted that treatments prioritizing visual feedback would result in higher pain relief based on the concept of sensory-motor incongruence (28). The concept of stochastic entanglement implies that treatments focusing on motor and somatosensory feedback, rather than visual feedback, would be more effective. Moreover, stochastic entanglement predicts that treatments focusing on physiologically appropriate motor control and somatosensory feedback can be effective regardless of visual feedback (note the conditional of “physiologically appropriate”). For instance, a blind amputee fitted with a highly integrated bionic limb controlled naturally, and receiving physiologically appropriate somatosensory feedback, would not suffer from PLP. Regarding non-invasive therapies using visual feedback, stochastic entanglement predicts that pain reduction would be independent of the level of anthropomorphic visual representation presented to the subject.

Upper limb amputees have been found to be more prone to suffer from PLP than lower limb amputees (47, 59). This observation could be explained by the difference in the amount of neural resources left susceptible to stochastic entanglement after amputation, as well as by the proportional degree of cortical reorganization and reduction of inter-hemispheric communication, but cannot be explained by sensory-motor incongruence. This observed difference regarding PLP incidence suggests a way to prevent the condition in the first place; namely, avoiding the neglect of the lost limb circuitry to reduce the probability of entanglement and maladaptive brain changes (cortical reorganization and reduced inter-hemispheric communication). Plasticity-based treatments described hereafter can be used to achieve this.

## Current treatment for PLP

Factors that modulate PLP are desirable targets for the development of therapies. Anecdotal accounts from patients suggest that changes in atmospheric pressure, temperature, or humidity influence the intensity of their PLP. However, scientific investigations have yet to confirm such observations. The scientific literature currently provides inconclusive evidence on amputation-induced functional and morphological changes in the brain that are also markers of PLP (29, 32, 39–42, 60–64), and therefore, causal or modulatory factors are far from reaching an established consensus.

Over 60 therapies for PLP have been proposed in the literature (23), but limited randomized control trials have been performed to provide high-quality evidence on their efficacy (65). Placebo effects are varied and often disregarded, even though they can account for more than the commonly cited 30% improvement (66). This is particularly important since treatments for PLP often report short-term relief of up to 30% (67). The following is a non-exhaustive summary of common treatments of PLP and their relation to the previously presented theoretical framework.

### Pharmacotherapy

Pharmacological approaches mostly address pain as a symptom, and therefore are limited to managing it, rather than curing it. Lidocaine has been found to reduced stump pain but not PLP (68, 69), supporting the aforementioned distinction between the underlying mechanism of these two different pains. Whereas pharmacological approaches have been largely successful at alleviating acute nociceptive pain, pharmacotherapy is currently considered unsatisfactory for chronic neuropathic pain (70). In addition, a major drawback of pharmacotherapy is the potential risk of addiction. To this end, Penfield strongly stated: “It is a major professional sin to allow a patient to become a drug addict if there is another solution” [preface to (71)]. This sentiment about reducing chronic pain at the cost of quality of life by utilizing opioids continues at the present (72).

### Surgical interventions

Re-amputation and neurectomy (resection of the neuroma) constituted some of the initial efforts to treat PLP, albeit unsuccessfully over the long-term (71). In the 1970s, non-surgical methods for the treatment of PLP were considered more effective than surgical ones (23). Recent development of surgical techniques such as target muscle reinnervation [TMR (16)] and regenerative peripheral nerves interfaces [RPNIs (17)] have shown promising results in reducing neuroma pain (73). However, evidence regarding the ability of these surgical techniques to relieve PLP is limited. Patients treated with TMR continue to report PLP (74), and no long-term data are yet available on the effect of RPNIs on PLP (17). It is worth reiterating that neuroma pain and PLP have different origins (nociceptive and neuropathic pain, respectively).

A neuroma can be mechanically stimulated by palpation, wearing a socket prosthesis, and contraction of stump muscles. If such actions predictably result in painful sensations, the source of the problem is likely a neuroma, and the *peripheral nociception* hypothesis accounts for such referred pain in the phantom. This also applies when the excitation is by chemical means or by infection (12, 13). Healthcare professionals are advised to first identify whether the source of PLP is a neuroma and, if so, provide treatment accordingly. For instance, TMR and RPNIs have been argued as relatively safe and effective surgical solutions for neuroma pain, which could be performed prophylactically to prevent neuroma formation at the time of amputation and potentially in cases of refractory neuromas in non-amputees (16, 17).

### Artificial limb replacement (prosthetics)

Restoration of motor function and sensory feedback via limb transplantation, regeneration, or prostheses would not only restore function, but would also alleviate PLP according to the aforementioned plasticity-based hypotheses. Limb regeneration is currently out of reach, and limb transplantation is limited. However, a new generation of highly integrated limb osseo-neuroprostheses that interface to bone, nerves, and muscles (75) could resolve PLP as they operate in daily life using direct sensory neural feedback (76). Preliminary findings from my research group in four subjects implanted with such technology indicate the absence of PLP (follow-ups from one to up to 5 years, unpublished data). However, controlled long-term studies are needed to provide high-quality scientific evidence concerning osseo-neuroprostheses' ability to ameliorate PLP.

Restoration of both motor and sensory function would be ideal. However, extensive use of simpler functional prosthesis, with no somatosensory feedback, is known to correlate with lower incidence of PLP (31). Based on this finding, Lotze et al. argued that extensive use of myoelectric prostheses prevents cortical reorganization, and thus prevents PLP. One must consider that muscles normally used for conventional myoelectric control are not the same as those used to produce the biological actuation. For example, in a transhumeral amputation, the biceps and triceps muscles are used to control the prosthetic hand, rather than intrinsic or even extrinsic hand muscles. Therefore, the cortical representation of the hand is not activated to control the prosthetic hand, at least not in its native functional organization, and therefore it is uncertain how control substitution would prevent cortical reorganization. Similarly, the inverse correlation between PLP and prosthetic use cannot be attributed entirely to resolving sensory-motor incongruence, as there is no intention of congruent phantom movement *per se*, but instead, control substitution. On the other hand, this common but unintuitive method of prosthetic control requires learning a new skill, for which the idle processing resources of the missing limb are likely recruited, thus potentially protecting them from a susceptible chaotic state in which they could entangle with the pain connectome (stochastic entanglement).

A degree of motor execution is certainly present in the PLP-prosthesis relation, as purely cosmetic prostheses do not seem to reduce PLP despite their anthropomorphic appearance (31). This suggests that motor control with its intrinsic feedback might be sufficient in most cases. In this regard, it is worthwhile to note that muscle contraction, even without joint actuation, produces non-negligible sensory feedback for contraction strength and muscle length. This proprioceptive feedback is used regularly by prosthetic users to fine-tune the intended strength of muscular contraction, which often translates to speed of prosthetic movement (proportional control). Prosthetic users rely on such intrinsic feedback while learning conventional myoelectric control. For example, using electrodes implanted on muscles (75), my research group was able to capture single motor action potentials and drive a prosthetic hand faster than the patient could perceive endogenous feedback from muscular effort. As a result, our patient reported the ability to actuate the prosthesis just by “thinking” about the movement. This perception arose arguably due to the lack of muscular feedback (the muscular effort component of proprioception), and was not appreciated by the patient, who preferred to feel muscular contraction in order to achieve better prosthetic control. The gain of his myoelectric amplifiers was therefore reduced, so a higher muscular contraction would be required to activate the prosthesis.

Patients treated with TMR who utilize a functional prosthesis, and yet still report PLP (74), pose a challenge to all plasticity-based theories. TMR allows the intuitive control of prosthetic limbs by using muscles at the stump as biological amplifiers of nerve signals that originally actuated the missing limb. In other words, subjects who undergo TMR utilize the original neural circuitry of the missing limb to control a prosthetic one. In a similar way, Targeted Sensory Reinnervation (TSR) can produce transfer sensations from the missing limb to the stump (77). Patients treated with targeted motor and sensory reinnervation have shown normalized primary motor and somatosensory cortices, and yet, there are reported cases of PLP (78). Sensory-motor incongruence and cortical reorganization are resolved in these patients, and purposeful use of the affected circuitry should disentangle it from the pain connectome as predicted by the stochastic entanglement hypothesis, yet PLP remains. A potential explanation could be the mismatch on neural reutilization. TMR uses hyper-reinnervation, meaning that a thick nerve is coapted to a considerably thinner one, thus only a fraction of the axons reinnervate the target. Furthermore, TMR in the upper limb typically allows for the control of up to three degrees of freedom, as opposed to the 27 available in an intact hand. This means that only a limited portion of the neural circuitry is back in use, and thus the degree of neural resources utilization might not be enough to disassociate from the pain circuitry. PME of the unrestored degrees of freedom (for example, wrist flexion/extension or finger control), would increase the plasticity required to potentially disassociate pain circuitry and thus alleviate PLP.

### Plasticity-based interventions

#### Motor imagery

Mental imagery of phantom movement has been reported to reduce PLP along with cortical reorganization (62). However, outcomes of controlled clinical trials concluded that motor imagery is ineffective (79, 80), and thus discouraged by its own (81). Nevertheless, motor imagery may still have a therapeutic role to play. In cases in which kinesiophobia is concurrent with PLP, motor imagery could be used as an initial treatment stage so other motor therapies can follow. Motor imagery is used currently in such a way as part of Graded Motor Imagery (GMI), a therapy model that consists in lateralization (right/left limb identification), motor imagery, and mirror therapy. This order of increasing task complexity is fundamental for the therapy's success (82). GMI has shown successful results in PLP and CRPS (83), although negative findings have been reported as well (84). Similarly graded approaches have been proposed, such as employing progressive muscle relaxation, motor imagery, and phantom exercises (85).

#### Mirror therapy

Mirror therapy is arguably the most common and cost-effective therapy for PLP and CRPS in clinical use. Introduced by Ramachandran and Rogers-Ramachandran (57), mirror therapy entails placing a mirror in eyesight of the missing limb, in order to reflect the movements of a contralateral and still available limb, while the subject is asked to perform parallel movements with both limbs. Originally using a mirror box, physical constrains could be eliminated with mirror glasses (86) or virtual reality (87). Reduced PLP along with normalization of cortical organization have been observed after mirror therapy (36). Although mirror therapy has shown successful results in controlled clinical trials on PLP (79) and CRPS (80), it has been argued that evidence supporting its success is insufficient (88) and largely anecdotal (58).

Mirror therapy was devised with the aim to provide anthropomorphic visual feedback (89), and visual feedback has been argued as the main reason for its therapeutic effect (58). Controlled clinical trials present conflicting evidence in this regard. Whereas Chan et al. found that no improvement was gained with a covered mirror (79), Brodie et al. found that visual feedback was not necessary for pain relief (90). These conflicting findings can be explained by the cortical reorganization and stochastic entanglement hypotheses, in which pain relief can be achieved by motor execution alone and not hindered by visual feedback. In contrast, sensory-motor incongruence is resolved only partially in mirror therapy as proprioceptive feedback is missing, and without visual feedback, no relief as reported by Brodie et al. (90) should be possible according to the sensory-motor incongruence hypothesis.

#### Sensory stimulation and discrimination

As noted previously, restoration of somatotopically appropriate sensory feedback alone could also be an effective therapy based on plasticity-based theories of PLP, excluding sensory-motor incongruence. In some patients, stimulation of the stump or face can produce referred sensations in the missing limb (phantom map). Simultaneous stroking of the phantom map and contralateral hand, while providing visual feedback by a mirror, has been reported to reduce PLP for short periods of time (91). Similar reduction of PLP has been observed when stimulating phantom maps located in the cheeks, while using virtual reality (VR) to provide a visual representation of the missing limb (92). However, phantom maps are often disorganized, incomplete, and relatively uncommon (30, 32). An alternative to produce somatosensory perception is the stimulation of afferent fibers, which requires the implantation of electrodes. In this regard, peripheral nerve stimulation has been reported to reduce PLP in a limited number of subjects (93–95). However, no long-term effect has been reported, and to date, no controlled randomized trials on direct nerve stimulation as a treatment for PLP have been performed.

Flor et al. propose a therapeutic approach in which patients learn to discriminate sensory stimuli at the stump (37). They showed that training on spatial or frequency discrimination increased acuity in the stimulated area and reduced PLP. Their subjects showed reversal of cortical invasion from the lip representation (lateral neighboring area), but did not study the stump representation where the stimulation took place. The possibility of relieving PLP by increasing sensory acuity at neighboring body parts was corroborated by Huse et al. (55). They showed enlargement of both neighboring body parts representations, arguably because both were stimulated. Enlarging cortical representation of the stump, by using control and sensory substitution, might be the cause behind the reduction of PLP when the idea of sensory discrimination was used to complement conventional myoelectric control (96). Whereas somatosensory appropriate stimulation engages the representation of the missing limb promoting preservation (reverse cortical reorganization), sensory discrimination at the stump enlarges the stump representation (purposeful cortical reorganization). Relief of PLP in the former but not the latter case agrees with the hypothesis of cortical reorganization, and both cases can be explained by disassociation based on the hypothesis of stochastic entangling.

#### Phantom motor execution-PME

**Figure 2:**
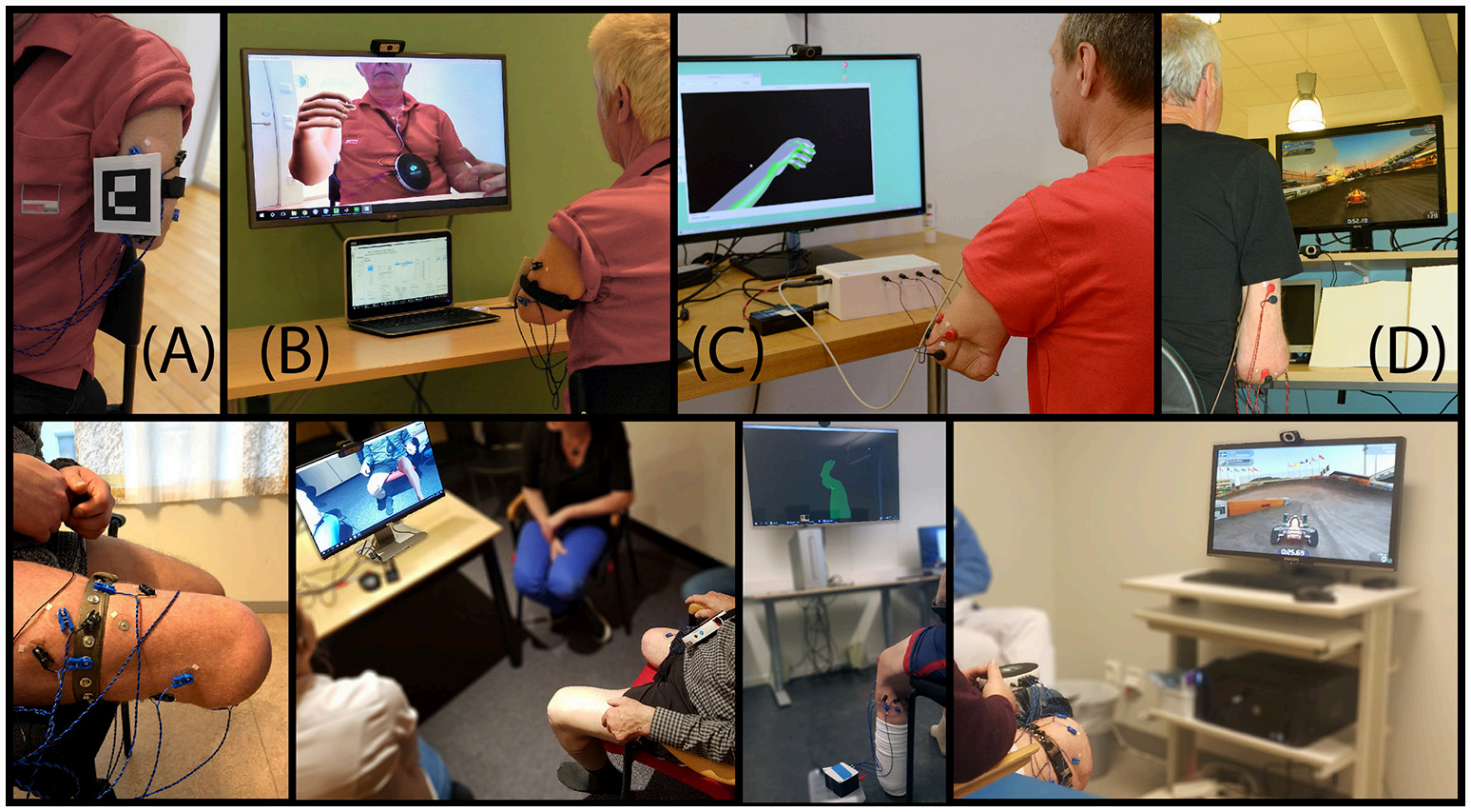
Phantom motor execution (PME) using myoelectric pattern recognition (MPR), virtual and augmented reality (VR/AG), and serious gaming (SG). A conventional treatment session of PME consists of identifying viable muscles at the stump, preferably as distally as possible, and placing skin surface electrodes on these muscles **(A)**. Targeted placement of electrodes is recommended but not necessary. In addition, a fiduciary marker is placed in sight of the webcam **(A)**. The subject is then instructed to follow the movements of a virtual limb, executing them as naturally as possible, while myoelectric activity is recorded. Algorithms used the collected information to train decoders to infer future intention of movement. Once the system has been trained, the subject can practice the execution of phantom limb movements in augmented **(B)** and virtual reality **(C, D)** environments with anthropomorphic **(B, C)** and non-anthropomorphic **(D)** visual feedback. Subjects provided written informed consent for the publication of these images.

Phantom motor execution (PME) entails producing phantom movements by recruiting the appropriate central and peripheral circuits, ultimately resulting in muscular activation at the stump (11). Mirror therapy could be used to facilitate PME. However, whether the subject engages in actual motor execution remains uncertain as motor output is not measured in any way. For instance, a subject could completely disregard movement in the lost limb and perform a full treatment focusing on the visual feedback provided by the contralateral limb only. In contrast, myoelectric decoding of motor volition at the stump ensures that movement execution is actually taking place. Muscular contraction is the ultimate physiological response to motor execution, and by extracting phantom motor intention from remaining muscular activity at the stump, one can ensure that the related central and peripheral circuitry is activated. The resulting phantom movement can then provide feedback to the user vis-à-vis by virtual or augmented reality, while taking advantage of serious gaming to maintain subject engagement throughout the therapy (97). This is the treatment modality considered for PME through this manuscript, namely myoelectric pattern recognition (MPR), virtual and augmented reality (VR-AR), and serious gaming (SG) (11, 97, 98), Figure 2.

## Working hypothesis on the mechanisms of phantom motor execution (PME)

I hypothesize that PME relieves pain by the following mechanisms:

- **Purposeful cortical reorganization**. PME treatment requires subjects to execute phantom movements as naturally as possible. “Natural movement” is explained as analogous to moving an able limb. Subjects are encouraged to perform bilateral movements, at least during the first few sessions, to aid in the understanding and performance of a natural movement. Regardless of their level of education, subjects are informed about the cortical reorganization findings and the stochastic entanglement hypothesis to stress the importance of “natural movements.” Subjects are told that the success of the therapy relies on them executing phantom movements as naturally as possible, as this would purposefully reengage the idle neural circuitry and potentially disentangle it from pain. Two effects are hypothesized to be at play at the cortical level during PME:
  1. Utilization of the original motor area corresponding to the missing limb would normalize it at the border with the face representation.
  2. Improved motor control of the stump musculature would enlarge its cortical representation into the missing limb area, as those neural resources are underutilized owing to the amputation.

In summary, the stump representation will invade, and likely overlap with, the original representation of the missing limb, whereas the opposite (lateral) border of cortical representation would be preserved owing to the reutilization of the missing limb circuitry.

- **Increased functional connectivity**. As previously noted, a correlation between reduced inter-hemispheric functional connectivity and PLP has been reported (40). It has been found that motor imagery does not result in spatial coupling, but rather, actual motor execution is required in order to achieve it (43). Therefore, by actually executing phantom movement, patients are likely to increase inter-hemispheric functional connectivity.
- **Undoing phantom paralysis**. Impaired phantom movement has been repeatedly found to be correlated with PLP (42, 99, 100). The majority of the subjects treated with PME reported to be unable to move their phantom limb at the first session. This became obvious when subjects were asked to produce phantom movements, to which they objected describing a paralyzed phantom. Subjects were persuaded to try to execute movements nevertheless, and eventually gained volition over their phantom limbs (11, 97, 98). At follow-ups, subjects commonly reported that the acquired skill to move their phantom seemed to help them to control pain episodes when this occurred outside the therapy session. This observation supports the aforementioned finding correlating phantom paralysis and PLP (42, 99, 100). This finding is mutually exclusive with sensory-motor incongruence as a potential cause of PLP. Observations by my research group on subjects treated with PME suggest that phantom movement without visual feedback seems to aid patients relieving their PLP, as opposed to exacerbating it as predicted by the sensory-motor incongruence hypothesis.
- **Competitive plasticity**. Neural processing resources in the brain are finite, meaning only a finite number of tasks can be processed at a given time. Neural networks occupied in a particular task are less likely to engage in the processing of another normally unrelated task. Conversely, neural networks deprived of their main function can engage in processing other less desirable tasks, such as pain perception. The challenge of producing myoelectric patterns different enough to control several distal movements engages a non-negligible amount of neural resources, potentially preventing them from contributing to pain processing. In summary, PME recruits susceptible neural resources, preventing their engagement in pain processing (competitive plasticity).

The PME hypothesis presented here can be tested by brain imaging (purposeful cortical reorganization and increased inter-hemispheric communication), and behavioral studies (phantom limb movement). My research group is beginning to test this hypothesis as part of a large international, double blinded, controlled clinical trial (101).

Based on the above mechanism hypothesized at play in PME, one can theorize that although visual feedback is required to reach the dexterity needed for competitive plasticity to be relevant, visual feedback does not need to be anthropomorphic. This prediction can be tested in a controlled trial in which visual feedback provided to the subject is either anthropomorphic or non-anthropomorphic, while keeping all the other aspects of PME constant.

The aforementioned mechanism for PLP treatment can also be used to prevent its development in the first place. PME soon after amputation could maintain inter-hemispheric communication, native cortical organization (or induced purposeful reorganization), and phantom limb movement. This would reduce the amount of susceptible neural circuitry, and thus reduce the probability of stochastic entanglement with pain. This prediction can also be tested by a controlled trial where PME is provided soon after amputation. The incidence of PLP in this group could be then compared to its natural occurrence, or as a result of providing another active treatment.

### Clinical findings on PME by MPR, VR/AR, and SG

PME using MPR, VR/AR, and SG was first evaluated in a patient with chronic intractable PLP in 2013 (97). At 72 years old, the subject was a male upper limb amputee who had suffered PLP for 48 years despite trying several medical and non-medical treatments. The patient reported a complex profile of PLP over time that motivated the development of a new comprehensive measure of pain considering intensity, time, and frequency, namely the weighted pain distribution (WPD). WPD has been found to correlate to conventional pain metrics, such as the numeric rating scale and the pain rating index (11). The subject reported a complete lack of phantom movement control, and perceived a static fist, described as strongly and stressfully clenched. He also reported low quality of sleep as high-intensity pain episodes would often awaken him during the night. PLP was gradually reduced to sporadic and short-duration pain episodes throughout 18 weekly sessions of treatment. In addition, PLP intrusion in sleep disappeared, and both the subject and his family reported this as a major benefit. The subject gained control over phantom limb movements, which he believes helped him to control the sporadic episodes of PLP. The patient was provided with a PME system (MPR, VR/AR, and SG) to use at home, and the treatment benefits have remained for over 5 years.

The above initial findings motivated a multi-center clinical trial on a similar patient population of chronic intractable PLP sufferers (11). Sixteen upper limb amputees with PLP for an average of 10 years, who tried all available treatment options at their clinics, were enrolled in four clinics and received 12 treatment sessions of PME. Pain was measured prior to each session in order to avoid misleading peaks of relief immediately after treatment. The subjects reported a gradual reduction of pain on the course of the treatment, which was measured at about 50% at the last treatment session. More than half of the patients reported a pain reduction of at least two points in the numeric rating scale (NRS). Pain reduction of 50%, or two points in NSR, is considered clinically relevant (102). Intrusion of PLP in sleep and activities of daily living was also reduced to about 50%, and half of the patients using medications reduced their intake by about 50%. These improvements were still observable 6 months after treatment, which is of paramount importance for the clinical relevance of treatments for chronic conditions (11).

Despite the considerations taken in the aforementioned clinical trial to avoid sources of bias, no control group was included and therefore confounding effects cannot be fully discarded (i.e., placebos). PME is currently under evaluation in an international (seven countries), double blind, randomized, controlled clinical trial (101). Both upper and lower limb amputees are enrolled in this multinational study. Preliminary results observed in lower limb PLP confirmed the feasibility of the approach in this patient population (98, 103).

PME in the proposed setup requires a conventional personal computer with a webcam, electromyography related electronics, and therapy guiding software formed by signal processing and machine learning algorithms, virtual and augmented reality environments, and games. This makes the technology portable and suitable for home use. Preliminary observations by my research group in four patients using such a system in their own at home indicate that outcomes comparable to those of a clinical environment can be attained (unpublished data).

Ramachandran et al. have reported that the perception of a phantom limb can disappear after mirror therapy, along with the pain that afflicted it (57, 89). Some patients aware of the possibility of such “phantom amputation” are hesitant to engage in treatments such as mirror therapy, as they do not desire their phantom to disappear. My research group and collaborators have not yet encountered said phantom limb disappearance after PME in over 30 subjects treated worldwide with follow-ups up to 5 years. On the contrary, gained movement skills over a vivid phantom limb has been the norm.

### Advantages of PME by MPR, VR/AR, and SG

Owing to the lack of standardization, mirror therapy allows patients to repeat the same movements inattentively. Simple and repetitive motor actions are insufficient to drive brain plasticity, particularly functional reorganization of cortical maps (104). It has been observed that sensory stimulation, without focusing on discrimination, does not result in a reduction of neuropathic pain (105). Brain plasticity requires mindful training. PME promoted by MPR forces patients to concentrate on producing distinct patterns of muscular activity, which remains challenging throughout the therapy by increasing the complexity of phantom movements (11, 97, 98). This increased dexterity and awareness of the stump musculature is hypothesized to drive “purposeful cortical reorganization.”

Purposeful cortical reorganization is ideally achieved by engaging both sensory and motor circuitry, but potentially also by engaging either independently, as noted before. PME is analogous to sensory discrimination in regards of the possibility to expand cortical representation of the stump (55), and the combination of both approaches would be potentially beneficial. Regarding engagement of the native missing limb circuitry, motor execution is practically advantageous over sensory feedback as subjects can engage in complex phantom movements, but eliciting rich sensations arising from the phantom limb would require the implantation of high-resolution neural interfaces, or the presence of phantom maps [naturally occurred or created by TSR (77)]. In other words, motor execution can still recruit the missing limb circuitry, as this is the result of a top-down, rather than a bottom-up process as in the case of sensory perception (the “bottom” part being biological sensors no longer available). Given the known involvement of the sensory cortex in motor control (56), PME execution appears as a more cost-effective solution in cases where restoration of both sensory and motor function is not feasible.

**Figure 3:**
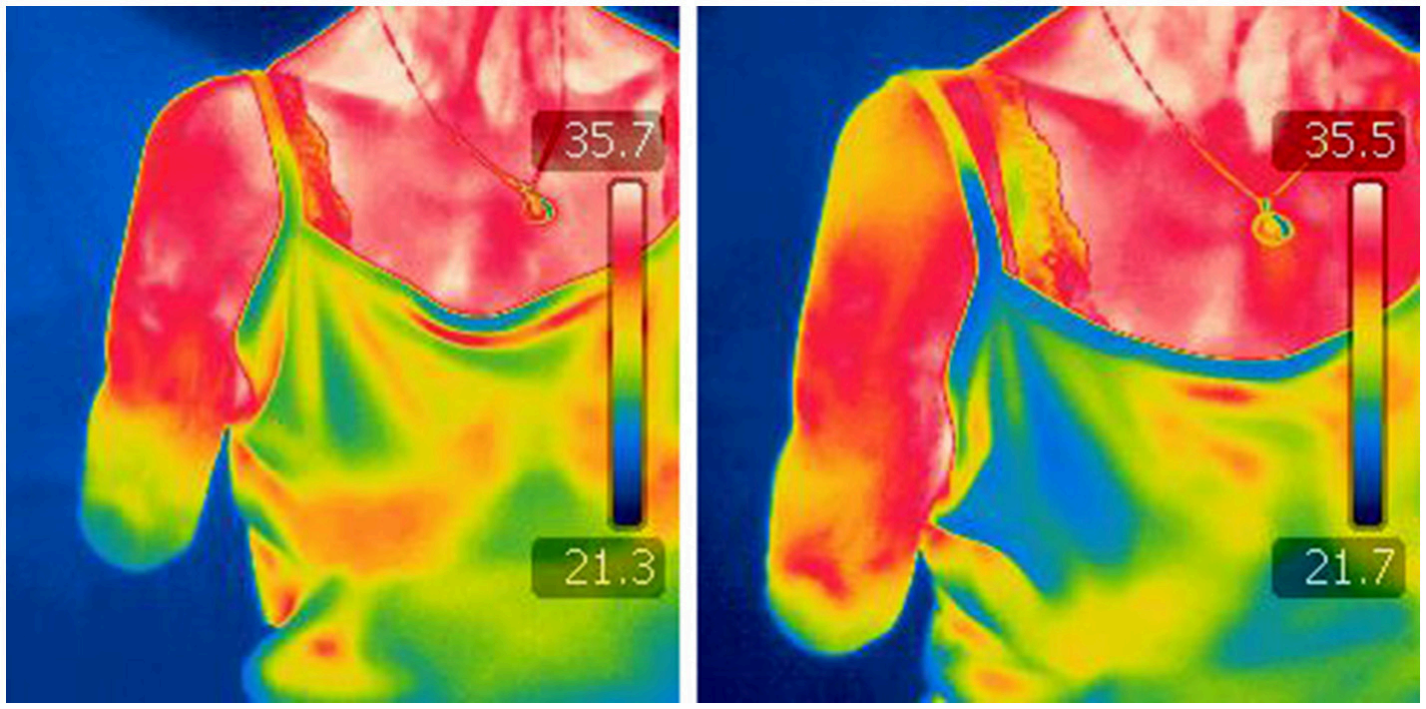
Infrared thermography before and after a PME treatment session. Images by a thermographic camera of the stump of a transhumeral amputee, before **(left)** and after **(right)** a session of Phantom Motor Execution (PME) using Myoelectric Pattern Recognition (PMR), Virtual, and Augmented Reality (VR/AR), and Serious Gaming (SG).

Intended movements of lost joints can be decoded using MPR despite the fact that available muscles at the stump did not originally actuate such joints. My research group has demonstrated that muscles above the elbow can be used to infer hand movements in transhumeral amputees (11, 75, 97), as well as that muscles above the knee can be used to decode foot movements in transfemoral amputees (98, 103). This is possible largely due to the synergistic activation of limb muscles during movement, and as such, decoding can be done in able-bodied subjects (98). In addition, MPR can gain access to motor information that previously reached the lost limb in case severed nerves naturally reinnervated stump muscles by peripheral sprouting (106). Conversely, information of distal movements remains inaccessible to motion tracking technologies. For instance, infrared sensors, or inertial measurement units, provide information of the position of the stump in space, and how remaining joints move around it, but they cannot inform about the intended action in the missing joints. Therapies employing such technologies cannot ensure the engagement of the affected motor circuitry, and therefore they are bound to provide limited pain relief when only focused on delivery of visual feedback (see section Theoretical framework on Phantom Limb Pain). A randomized controlled clinical trial on mirror therapy found that using augmented reality, instead of a conventional mirror, had no effect in pain relief (107). This result can be explained by the fact that the contralateral limb, rather than the affected limb, was used as the source of control and therefore PME was not guaranteed. PME using MPR ensures the activation of motor circuitry down to the stump, which in addition to addressing maladaptive plasticity down to the spinal cord, can also temporally normalize the stump temperature due to muscular contractions (Figure 3).

**Figure 4:**
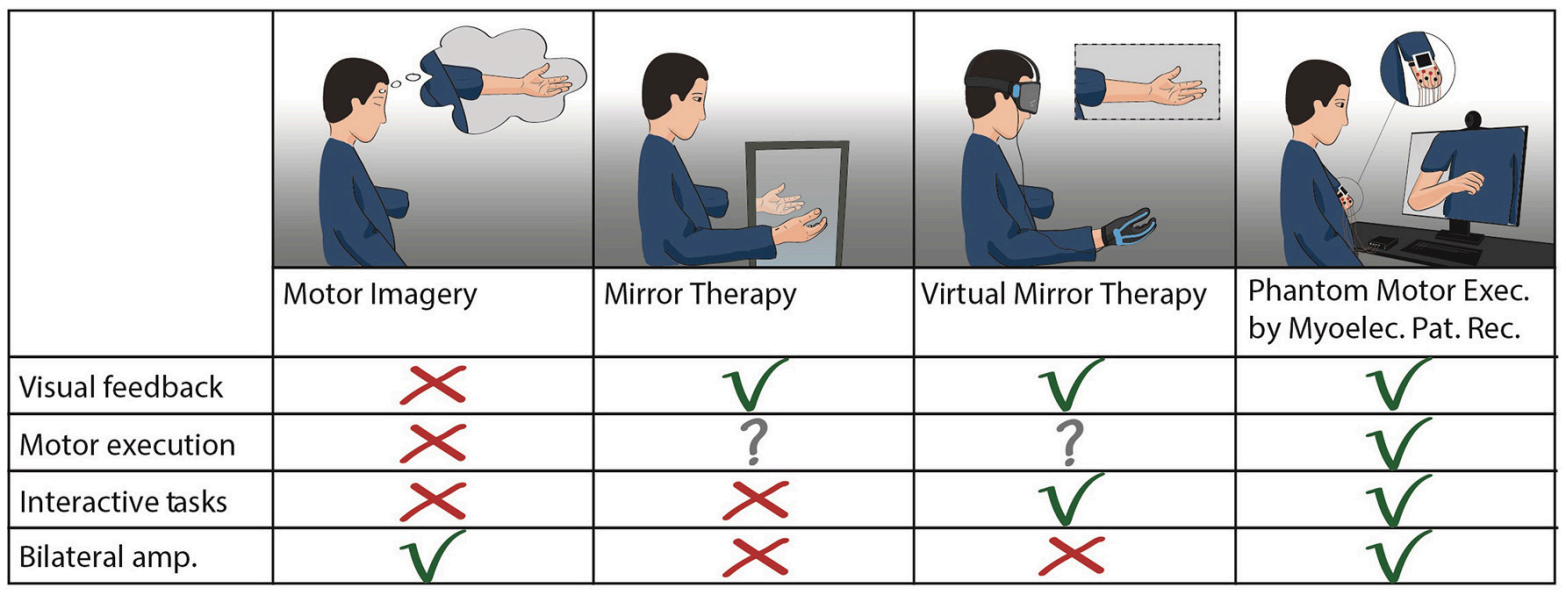
Treatments for PLP based on motor control. Comparison between plasticity-based treatments using motor imagery or execution of phantom limb movements. Mirror therapy and virtual mirror therapy fundamentally differ only in the source of visual feedback (analog or digital). Virtual mirror therapy illustrates the cases where a functional contralateral limb is the source of control for the virtual limb, as in mirror therapy. Phantom motor execution is illustrated as used with myoelectric pattern recognition and augmented reality. Virtual and augmented reality, as well as serious gaming, can be implemented in both virtual mirror therapy and phantom motor execution.

The stump and phantom limb could be further neglected if a plasticity-based therapy relies on the contralateral limb. Using instrumented gloves, or any technology worn or requiring the contralateral limb (Figure 4), makes the approach equivalent to mirror therapy from the mechanistic viewpoint. In addition, such an approach restricts its application to unilateral amputees with a functional remaining limb, albeit that it has been suggested that a third-person's limb might be used to overcome this problem (108). Overall, technologies that disregard the missing limb would result in a more complex and expensive setup than using a conventional mirror, although not necessarily more effective beyond the placebo effect brought about by sophisticated technology (expectation).

### Limitations of PME by MPR, VR/AR, and SG

PME could be hindered by neuropathies that prevent the subject from producing motor control, such as motor extinction (109). Some neuropathies at the cortical level could be overcome by using direct current transcranial stimulation (tDCS) (110), or transcranial magnetic stimulation (TMS) (111), to facilitate motor execution (112). Pan et al. showed that amputees under tDCS increased their ability to produce different patterns of myoelectric activity related to phantom movements (112), and therefore tDCS during PME therapy might accelerate PLP relief.

A contraindication of PME is the presence of stump pain or neuromas that become stimulated during muscular contractions. This is because stump contractions would be painful, and thus the PME treatment session would be painful as well. However, patient and physician should decide the level of stump pain at which PME becomes inappropriate. In a single-case study, a transradial amputee was treated with PME despite suffering considerable stump pain. This was owing to the subject's insistence on trying PME after exhausting all other clinical alternatives. The subject considered that his level of pain was high and constant, and therefore a small increase during the therapy was irrelevant. After 13 weekly sessions of 2 h of PME, PLP was reduced from 9 to 3 NRS, and stump pain was reduced from 10 to 3 NRS. The reduction of stump pain was unexpected but arguably related to the overall perception of both pains (113). Owing to this single positive outcome, clinicians should be cautious when considering patients with significant stump pain as candidates for PME treatment.

The obvious limitation of using MPR is the need of volitional control over stump muscles. Although limited musculature is required, there must be present at least portion of biceps and triceps brachii muscles in the upper limbs, or quadriceps and hamstrings muscles in the lower limbs. MPR is not ideal in subjects with shoulder or hip disarticulation unless they undergo a surgical intervention such as TMR. Similar limitations apply to subjects with excessive soft tissue, and those who suffered nerve injuries such brachial plexus avulsion. In case of uncertainty, an evaluation using MPR is recommended to determine if myoelectric activity can be recorded and whether it is usable for MPR.

## The lure of VR and other emerging technologies

Developing sophisticated technologies for the treatment of a given condition requires time, effort, and financing. In addition, there is an opportunity cost once a given approach has been selected, and such cost might be considerable if technologies are chosen with an under-informed basis. A word of caution is therefore pertinent on the matter, as novel therapies found in the literature often provide unclear mechanistic bases.

Several approaches to relieve PLP using VR had been reported in the literature (114), and visual feedback is often cited as the main reason for pain relief (57, 58). However, there is limited evidence supporting the high importance so far given to anthropomorphic visual feedback. Although alterations to the visual representations of a limb have been reported to modify pain perception (115, 116), a recent systematic review by Boesch et al. found limited evidence to support the argument that bodily illusions can alter pain (117). Furthermore, in the cases where PLP is maintained by maladaptive changes at the spinal cord, visual input is unlikely to affect such circuitry and therefore be directly responsible for PLP relief. One can make the case that visualization of healthy limbs alone is clearly not sufficient to relinquish PLP, as sufferers observe healthy limbs in their daily life, and yet PLP prevails. Similarly, mirror therapy would be successful in all cases if only anthropomorphic visual feedback would be required to relinquish pain. If the main reason for using digital VR is to provide a realistic limb representation, one should consider utilizing a mirror instead. The similarity and fluidity of movement in mirror image is a good as it could be, at a fraction of the cost.

Head-mounted displays (HMD) are often argued as preferable due to the higher immersion provided by a first-person perspective, but again, limited support exists for the idea that immersion, or a first-person perspective, mediate neuropathic pain beyond serving as a distraction. The use of more sophisticated technologies, such as HMDs, can increase the therapy's appeal and although this is important, it does not necessarily increase the therapy's efficacy beyond creating an initial incentive for patients to use it. In this regard, it is worth noticing that pain itself is already a strong incentive to adhere to therapy.

Realistic virtual environments are often sought to promote embodiment, which is believed to help in the relief of pain. This line of thought has two problems. Firstly, embodiment is mainly comprised of agency and ownership. Agency is not related to the virtual representation, but to the perceived control over such representation. This leaves only the ownership component of embodiment to visual feedback. However, visual feedback alone does not induce ownership, but requires synchronized and somatotopically congruent tactile feedback as well. That is, a visual stimulation of the virtual index finger must correspond in time and location to a tactile perception in the index finger. This presents a problem in amputees, as distally referred tactile sensation can only be achieved non-invasively with the presence of a phantom map [naturally occurred or created by TSR (77)], or invasively via direct nerve stimulation. Secondly, even if ownership can be achieved, there is not yet strong scientific evidence to support the idea that embodiment mediates PLP. Embodiment of limb prostheses has not been correlated to absence of PLP (118), nor has perceived ownership of a rubber hand (as in the rubber hand illusion) demonstrated pain relief (119). The analgesic effects of embodiment, or that of a realistic visual representation of the missing limb, are poorly supported by scientific evidence as of today, and thus should not be used as the sole argument to support novel PLP treatments. Further research on the mediation of pain by the aforementioned aspects is required for these to become scientifically sound targets for pain treatment. Lastly, VR is commonly misunderstood as a therapy in and of itself, rather than a tool that is used for the design of interventions. This is an important distinction because the success of any given therapy is less dependent on the technology employed, than on how such technology is applied.

## Conclusion

This article presented a theoretical framework for Phantom limb pain (PLP) and two working hypotheses for its origin and treatment, respectively. Implications, predictions, and experiments to test the validity these ideas were described. Ongoing experiments will further support or challenge the ideas of stochastic entanglement and phantom motor execution presented here. PLP is a complex condition that requires careful evaluation. Distinction between nociceptive and neuropathic sources of the referred painful sensations is necessary for prescription of suitable treatments. Similarly, novel treatments must consider current clinical and neuroscientific finings to improve their chance of success. In this regard, controlled randomized trials and long-term follow-ups are necessary to identify truly effective therapies.

## Author contributions

The author confirms being the sole contributor of this work and approved it for publication.

### Conflict of interest statement

MO-C was partially funded by Integrum AB, a for-profit organization that might commercialize a device for PLP treatment based on the concepts described in this article. MO-C has made the core technology for such device freely available as open source (machine learning, virtual reality, and electronics).
